# Trained Immunity Induced by Oxidized Low‐Density Lipoprotein Is Dependent on Glutaminolysis

**DOI:** 10.1096/fj.202500802R

**Published:** 2025-06-27

**Authors:** Alice Scarpa, Yerin Jung, Arslan Hamid, Vasiliki Matzaraki, Tushar More, Alexander Heinz, Laszlo Groh, Siroon Bekkering, Karsten Hiller, Leo A. B. Joosten, Mihai G. Netea, Katarzyna Placek, Niels P. Riksen

**Affiliations:** ^1^ Department of Immunology and Metabolism, Life and Medical Sciences (LIMES) Institute University of Bonn Bonn Germany; ^2^ Department of Internal Medicine Radboud University Medical Center Nijmegen the Netherlands; ^3^ Department of Biochemistry and Bioinformatics Technische Universität Braunschweig Braunschweig Germany

**Keywords:** atherosclerosis, glutamine, glutaminolysis, Krebs cycle, oxLDL, trained immunity

## Abstract

Atherosclerosis is a chronic inflammatory disease of the arterial wall that causes cardiovascular disease. Monocyte‐derived macrophages are an important contributor to atherogenesis. Monocytes can become primed for higher responsiveness to secondary, unrelated stimuli—a phenomenon known as trained immunity—a process driven by intracellular metabolic and epigenetic reprogramming. Oxidized low‐density lipoprotein (oxLDL) induces trained immunity by enhancing glycolysis and oxidative phosphorylation (OXPHOS). Glutamine is known to enter the Krebs cycle through glutaminolysis where it can be used for ATP synthesis via OXPHOS. We therefore explored the role of the glutaminolysis pathway in oxLDL‐induced trained immunity. Primary human monocytes from healthy donors were exposed to oxLDL for 24 h, followed by differentiation into macrophages over 6 days in culture medium. Thereafter, cytokine production capacity was assessed by stimulating them with Toll‐like receptor agonist. Co‐administration of the glutaminase inhibitor CB‐839 during oxLDL exposure reduces glutamine anaplerosis. This prevented oxLDL‐induced trained immunity, with diminished cytokine production capacity, associated with a reduced oxygen consumption rate (OCR), and glycolysis rate (ECAR). The role of glutaminolysis for induction of trained immunity was validated genetically, by showing significant associations between several single‐nucleotide polymorphisms in genes related to glutaminolysis and ex vivo cytokine production in oxLDL‐trained monocytes from 243 healthy volunteers. Finally, we identified a positive correlation between glutamate and Krebs cycle metabolites with inflammatory circulating biomarkers and monocyte counts in an independent cohort of 302 obese individuals. Altogether, these data suggest a crucial role of glutaminolysis in the establishment of oxLDL‐induced trained immunity.

## Graphical abstract

A brief exposure to oxLDL induces a trained immunity phenotype in monocytes, characterized by increased cytokine production upon secondary LPS challenge. This enhanced proinflammatory phenotype is supported by increased glutaminolysis and elevated glutamate processing through the TCA cycle, as well as increased oxidative phosphorylation. Blocking the glutaminase enzyme prevents the establishment of this phenotype. The graphical abstract was created with BioRender.com.

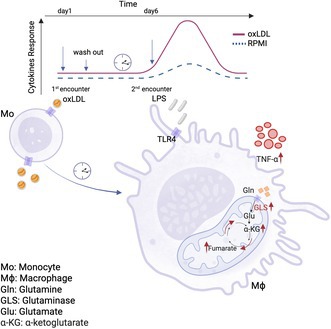

## 1 Introduction

Despite advancements in treating stroke and myocardial infarction, cardiovascular disease (CVD) remains a leading cause of death worldwide [1]. The underlying cause of CVD is a buildup of atherosclerotic plaques in the inner lining of arteries. Immune cells, especially monocytes and macrophages, play a pivotal role in atherogenesis [2]. Monocytes are recruited to activate endothelial cells and enter the intimal layer, where they differentiate into macrophages. These cells can ingest oxidized lipoproteins to become cholesterol‐laden foam cells. They also produce proinflammatory mediators such as cytokines, chemokines, and reactive oxygen, contributing to the inflammatory process and progression of atherosclerosis.

Trained immunity, also called innate immune memory, has been recently identified as a novel mechanism contributing to atherosclerosis development [3]. Trained immunity is induced by certain microorganisms or microbial ligands such as Bacille Calmette Guérin, or β‐glucan [4] and results in a long‐term functional adaptation of innate immune cells, leading to enhanced responses to subsequent unrelated inflammatory challenges [5]. However, this phenotype can also be inappropriately triggered by endogenous atherogenic molecules, like oxidized low‐density lipoprotein (oxLDL) [6], uric acid crystals [7], or catecholamines [8]. Recent murine studies unequivocally showed that high fat diet‐induced or hyperglycemia‐induced trained immunity can accelerate atherosclerosis [9, 10].

OxLDL is a modified lipoprotein that plays a critical role as an atherogenic molecule within plaques [11]. We have previously shown that brief exposure of monocytes to physiologically relevant concentrations of oxLDL drives the development of a hyperresponsive trained macrophage phenotype, with an augmented production of atherogenic cytokines, chemokines, and matrix metalloproteinases, when they are restimulated [6]. This enhanced functional state results from epigenetic and metabolic rewiring of the innate immune cells [12]. The development of trained immunity after exposure to BCG, β‐glucan, and oxLDL is dependent on metabolic pathways such as glycolysis, oxidative phosphorylation, and the mevalonate synthesis pathway [4, 13, 14]. An additional pathway that is critical for β‐glucan‐induced trained immunity is glutaminolysis and subsequent replenishment of the Krebs cycle [4, 12]. This results in the accumulation of metabolites, such as fumarate, that modulate the activity of epigenetic enzymes and therefore contributes to the epigenetic rewiring [4]. It is currently unknown whether this pathway also contributes to oxLDL‐trained immunity. Given the role of trained immunity in the development of atherosclerosis, this knowledge could offer new potential treatment targets.

The present study hypothesized that the conversion of glutamine into intracellular glutamate is important for the establishment of oxLDL‐trained immunity. By using pharmacological and genetic strategies, we demonstrated in a well‐established in vitro model of trained immunity [15, 16] that oxLDL‐induced trained immunity is critically dependent on glutaminolysis.

## 2 Materials and Methods

### 2.1 Oxidized‐LDL Preparation

OxLDL was prepared from LDL as described previously [17]. LDL was isolated from the serum of healthy subjects via ultracentrifugation. Then, LDL was oxidized with 20 μmol CuSO_4_/L for 16 h in a Thermo‐shaker at 400 rpm at 37°C. OxLDL was then dialyzed with PBS (w/o Calcium and Magnesium), and protein concentration was determined using the Pierce BCA Protein Assay Kit (Thermo Scientific, Pierce BCA Protein Assay Kits) following the manufacturer's instructions.

### 2.2 Monocytes Isolation and In Vitro Induction of Trained Immunity

Monocytes were isolated from the buffy coats of blood of healthy donors from the blood bank of the University Hospital Bonn. The study was approved by the local ethic commission (ethical vote# 009/21). OxLDL‐trained immunity in vitro protocol of adherent monocytes was performed as described previously [15]. Briefly, adherent monocytes were seeded at 100.000 cells/well in a 96‐well plate (Corning) in RPMI 1640 Dutch modified medium (Invitrogen) supplemented with 10% pooled human serum, 2 mM glutamax (Gibco) and 1 mM sodium pyruvate (Gibco), 50 μg/mL gentamicin (Thermo Scientific) and stimulated with 10 μg/mL of oxLDL for 24 h. In the experiments with pharmacologic inhibitors, cells were pre‐incubated for 1 h with 50 μM CB‐839 (MedChem Express) and 10 μM V9302 (MedChem Express) before oxLDL treatment. Inhibitors were dissolved in DMSO according to the manufacturer's recommendation. RPMI containing DMSO was always included as a control. The medium was changed after 24 h, and cells were left to differentiate for 5 days in the completed RPMI 1640 medium. On day 6, cells were restimulated with the medium or 10 ng/mL LPS (serotype 055:B5, Sigma Aldrich, St. Louis, MO, USA and further purified as described previously [18]). After 24 h, the supernatant was collected and used for cytokine production assay.

Cytotoxicity of pharmacological inhibitors was tested by measuring lactate dehydrogenase in the conditioned media following a 24‐h incubation period according to the manufacturer's instructions (CytoTox96, Promega) (Figures S2 and S3).

### 2.3 Apoptosis Assay

On day 6 of trained immunity protocol macrophages were collected using cell scraper and cold PBS and stained with FITC conjugated Annexin V (R&D System) and Live/Dead staining (Biotium, Live‐or‐Dye Fixable Viability Staining Kits), following the manufacturer's instructions. Cells were stained for necrotic cells with Live/dead staining for 10 min at 4°C and then with FITC Annexin V for 15 min at RT. Cells were analyzed by FACS (BD LSR II), and the percentage of necrotic and apoptotic cells was calculated and data were visualized using FlowJo version 10 (FlowJo LLC).

### 2.4 Seahorse XF Extracellular Flux Analyzer

Approximately 1 × 10^7^ monocytes were trained with oxLDL (10ug/mL) in the presence or absence of the CB‐839 inhibitor in 10‐cm Petri dishes (Sarstedt) for 24 h. After this period, monocytes were washed with warm PBS and incubated in a normal culture medium with 10% human pooled serum at 37°C, 5% CO2 for the following 5 days. At day 6 of the culture, cells were detached with cold PBS and centrifuged for 10 min at 300 *g* at 4°C, and 1 × 10^5^ cells were seeded in replicates in a 96‐well plate (Agilent). About 60 min before the run, the medium was replaced by Seahorse XF DMEM Medium pH 7.4 with 5 mmol/L glucose, 1 mmol/L pyruvate, and 2 mmol/L L‐glutamine and incubated for 1 h in a non‐CO2 incubator at 37°C. Oxygen consumption rate (OCR) and extracellular acidification rate (ECAR) were measured according to the Seahorse XF Cell Mito Stress Test conducted in the Agilent Seahorse XF Analyzer. The following compounds were injected during the test: 1 μmol/L oligomycin (Sigma Aldrich #A4876), 1 μmol/L fluoro‐carbonyl cyanide phenylhydrazone (FCCP) (Sigma Aldrich, #C2920), and 1.25/2.5 μmol/L rotenone/antimycin A (Sigma Aldrich #R8875, #A8674).

### 2.5 Cytokine Measurement

The concentration of the proinflammatory cytokine in macrophage supernatants was measured using DuoSet ELISA kit for human TNFα (#DY210, R&D) following the manufacturer's instructions. The absorbance was quantified at 450 nm using a Tecan reader. Concentrations were calculated by 4PL parameters logistic regression using GraphPad Prism.

### 2.6 Genetic Analysis

Genotyping was performed on 243 healthy individuals of Western European ancestry from the 300BCG cohort (NL58553.091.16) using the commercially available single‐nucleotide polymorphism (SNP) chip (Infinium Global Screening Array MD v1.0) from Illumina. In parallel, PBMCs of each donor were stimulated ex vivo with oxLDL according to the in vitro trained immunity protocol. Cytokine concentrations were measured in the supernatants by ELISA. The methods for QTL mapping have been described previously [19]. Ethical approval of the cohort studies was granted by the local Ethics Committee (CMO regio Arnhem‐Nijmegen; numbers 2011/399 and NL58553.091.16) [20].

### 2.7 Cell Culture With Stable‐Isotope Tracing

Roughly 1 × 10^7^ adherent monocytes were seeded in 10 cm Petri dishes (Sarstedt) following the in vitro oxLDL‐induced trained immunity protocol in the presence or absence of the CB‐839 inhibitor. The cells were cultured in 10 mL culture medium containing the stable‐isotope tracer L‐glutamine (13C5, 99%, ref.: CLM‐1822‐H‐0.25, Euroisotope, Cambridge Isotope Laboratories, USA) for 24 h at 37°C, 5% CO2. Following tracer injection in the media, 24 h before cell collection, at day 1, day 3, and day 6 of culture, metabolites were extracted as described below.

### 2.8 Metabolite Extraction and GC–MS Measurement

Metabolites were extracted from cells following a previously described protocol [21]. In summary, cells were washed with 0.9% NaCl and quenched with ice‐cold methanol and ice‐cold ddH2O containing 1 μg/mL D6‐glutaric acid as an internal standard. After scraping, cell extracts were transferred to tubes containing ice‐cold chloroform. The mixtures were vortexed at 1400 rpm for 20 min at 4°C, followed by centrifugation at 17 000 *g* for 5 min at 4°C to facilitate phase separation. A 300 μL aliquot of the upper polar phase was transferred into GC glass vials with micro inserts and dried under vacuum at 4°C. Relative metabolite levels and isotopic enrichment were analyzed via GC–MS as previously reported [21, 22]. The dried extracts were derivatized with methoxylamine (20 mg/mL in pyridine) and MTBSTFA before injection into the GC–MS system. Metabolite separation was conducted using an Agilent 7890B gas chromatograph with a 30 m DB‐35 ms and 5 m Duraguard capillary column. Detection was performed in either full scan or selected ion mode using an Agilent 5977 MSD system. Chromatograms were analyzed, and mass isotopomer distributions and relative metabolite comparisons were calculated using the Metabolite Detector software [23].

### 2.9 Data

The gene expression datasets used in this study were available in the Gene Expression Omnibus, series number: GSE166238 [24], and in the ArrayExpress database at EMBL‐EBI (https://www.ebi.ac.uk/arrayexpress), series number E‐MTAB‐9399 [25]. Dataset for the 300‐OB cohort is publicly available at the Human Functional Genomics Project (HFGP) website and can be accessed at https://hfgp.bbmri.nl/.

### 2.10 Gene Signature Identification (RNA‐Seq)

Differentially expressed genes (DEGs) were identified and used for pathway enrichment analysis. Kyoto Encyclopedia of Genes and Genomes (KEGG) pathway analysis was conducted to identify significantly enriched pathways using the clusterProfiler package in R, with an adjusted *p*‐value cutoff of 0.1 for significance. Visualization of the Alanine, aspartate, and glutamate metabolism pathways (hs00250) was performed using the pathview package. Gene expression values were mapped onto the pathway diagram. Expression limits were set at +1 and −1 to define thresholds for upregulation and downregulation.

### 2.11 Differential Gene Expression Analysis (RNA‐Seq)

Bulk RNA sequencing data were analyzed using DEGs. Normalization was done for library size and composition bias using the TMM method implemented in the *edgeR* package as it offers robust normalization for downstream differential expression analysis, which was performed using the R package, *DESeq2*. For statistical testing, a negative binomial generalized linear model was used, and genes with an absolute log2 fold change of more than 1.5 and a false discovery rate (FDR)‐adjusted *p*‐value less than 0.1 were considered significantly differentially expressed.

The volcano plot was constructed to represent DEGs, log2 fold‐change values were placed on the x‐axis, while on the y‐axis there were log10‐transformed adjusted *p* values. According to the threshold set, the genes were considered upregulated, downregulated, and insignificant. Plotting was done using R, using *ggplot2*. Color coding discriminated between the up‐ and downregulated genes.

### 2.12 Statistical Analysis

Statistical analyses were performed using GraphPad Prism.

Wilcoxon signed‐rank test and Mann–Whitney test were used to compare the differences among experimental groups. A *p*‐value < 0.05 was considered to be statistically significant. Data represent mean ± SEM.

Spearman's rank correlation coefficient was used to evaluate the relationships between plasma metabolites, inflammatory markers, and blood cell counts, accounting for potential non‐linear associations. Multiple comparisons were controlled using the Benjamini–Hochberg procedure, and statistical significance was denoted as follows: * (FDR‐adjusted *p* < 0.05), ** (*p* < 0.01), *** (*p* < 0.001), and **** (*p* < 0.0001). Heatmaps were generated using the *pheatmap* package in R, with hierarchical clustering applied to group variables based on correlation patterns. All statistical analyses were conducted in R, using the stats package for correlation calculations and the *p*.adjust function for FDR correction. Correlation matrices were visualized with red and blue, indicating positive and negative correlations, respectively, and significant correlations were highlighted with asterisks.

## 3 Results

### 3.1 Metabolism and Immune‐Related Genes Are Upregulated in oxLDL‐Exposed Monocytes

**FIGURE 1:**
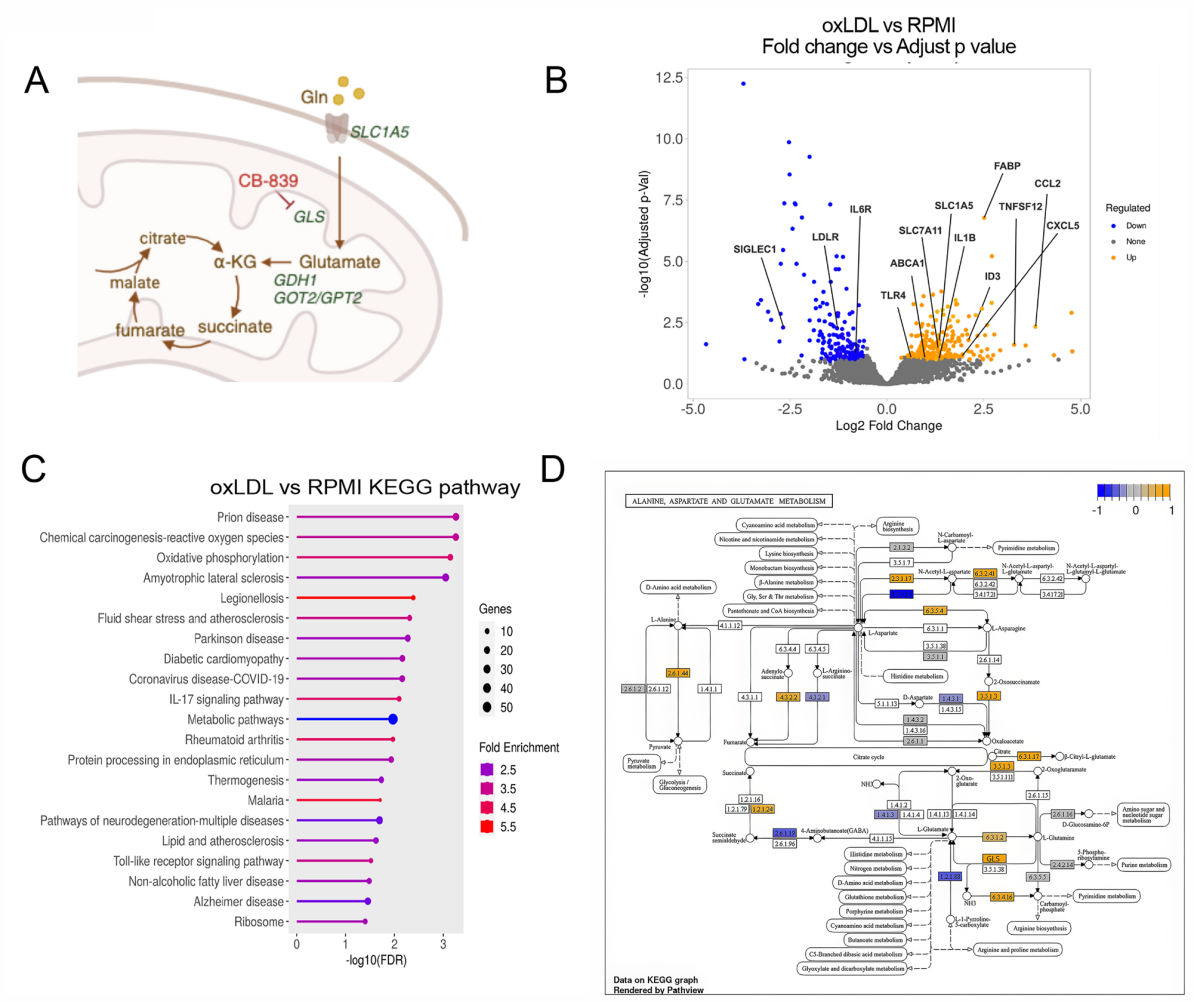
OxLDL‐exposed monocytes have an upregulation of metabolism and immune‐related genes. (A) A schematic representation of glutamine processing within the cells. (B) The volcano plot illustrates the differentially expressed genes in monocytes after exposure for 24 h to oxidized low‐density lipoprotein (oxLDL) compared to the control situation of exposure to only RPMI medium. Genes relevant for atherosclerosis and glutamine metabolism are named. Log 2‐fold change of 1.5 and *p*‐value of 0.05, adjusted *p*‐value of 0.1 (*n* = 3 individuals). (C) KEGG pathway analysis of differentially regulated genes in oxLDL‐exposed vs. RPMI‐exposed monocytes. Top 20 KEGG terms with *p*adj < 0.1. (D) Pathview of KEGG analysis shows alanine, aspartate, and glutamate pathways; in yellow are represented upregulated genes and in blue downregulated genes within monocytes following 24 h of exposure to oxLDL. GLS enzyme is named. αKG, alpha‐ketoglutarate; ABCA1, ATP binding cassette subfamily A member 1; ASCT2/SLC1A5, solute carrier family 1 member 5; CCL2/MCP‐, CC‐motif chemokine ligand 2; FABP4, fatty acid binding protein 4; GLS, glutaminase; GLUD1, glutamate dehydrogenase; GOT2, glutamic‐oxaloacetic transaminase 2; GPT, glutamate‐pyruvate transaminase; IL6R, interleukin 6 receptor; LDLR, low‐density lipoprotein receptor; LDLR, low‐density lipoprotein receptor; SIGLEC1, sialic acid binding Ig like lectin 1; SLC7A11, solute carrier family 7 member 11; TLR4, toll‐like receptor 4; TNFSF12, TNF superfamily member 12.

To explore changes in metabolic pathways in oxLDL‐induced trained immunity (Figure 1A), we analyzed the publicly available RNA‐seq dataset (Gene Expression Omnibus, series number: GSE166238) [24] of human primary monocytes exposed to oxLDL for 24 h compared to cells only exposed to the culture medium (RPMI). Our analysis revealed that the transporter for neutral amino acid and glutamine Solute Carrier Family 1 Member 5 (*SLC1A5/ASCT2*) was overexpressed in oxLDL‐treated monocytes vs. untreated cells (Figure 1B). Furthermore, genes implicated in inflammation and chemotaxis, including the Toll‐like receptor 4 (*TLR4*) and CC‐motif chemokine ligand 2 (*CCL2*) [6] and genes involved in atherogenesis, including fatty acid binding protein 4^27^ and ATP binding cassette subfamily A member 1 (*ABCA1*) [26, 27], were also upregulated by oxLDL treatment. The KEGG analysis revealed metabolic pathways, and in particular oxidative phosphorylation (Figure 1C), as well as glutamine‐related processes such as glutathione metabolism and purine nucleotide biosynthetic process to be enriched in upregulated genes (data not shown). Furthermore, the alanine, aspartate and glutamate pathways showed an overexpression of glutaminase (*GLS*) enzyme, which converts glutamine to glutamate, in oxLDL‐treated monocytes in comparison to the control (Figure 1D). In addition, *GLS isoform 2* (*GLS2*) enzyme was upregulated in the multipotent progenitor population (MPP) in patients with coronary artery disease (CAD) compared to patients without atherosclerosis (Figure S1) [25, 28]. Altogether, the transcriptomic data suggest the upregulation of glutaminolysis in oxLDL‐exposed monocytes.

### 3.2 Glutaminolysis Inhibition by CB‐839 Prevents Trained Immunity Induction by oxLDL

**FIGURE 2:**
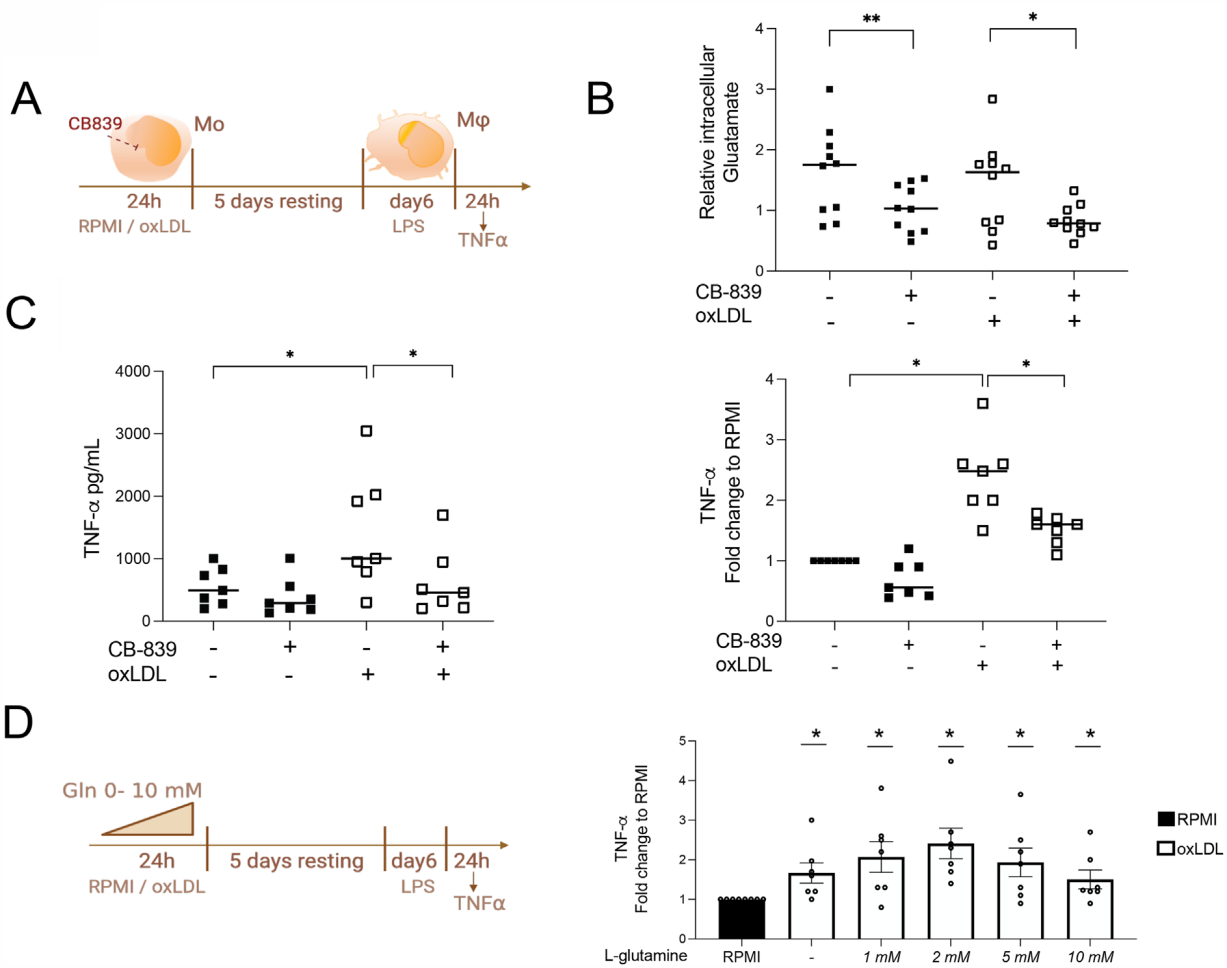
Glutaminolysis inhibition by CB‐839 prevents trained immunity induction by oxLDL. (A) Schematic representation of the in vitro design of oxLDL‐induced trained immunity with the GLS inhibitor CB‐839. (B) Intracellular glutamate concentration measured after the first 24 h of exposure as indicated in A. by GC–MS. The data are shown as mean + SEM, *n* = 10, *p < 0.05, **p > 0.01 Wilcoxon signed‐rank test. (C) left: TNF‐α concentration measured by ELISA in macrophage cultures treated as shown in A after 24 h restimulation with LPS. Right: Fold‐change TNF‐α to vehicle (DMSO). Median + SEM, *n* = 6/7, *p < 0.05, Wilcoxon signed‐rank test. (D) L‐glutamine supplementation during in vitro trained immunity model. Left: Schematic representation of the in vitro treatment. Monocytes were cultured with medium alone (RPMI) or oxLDL and incubated with different concentrations of L‐glutamine (in a range from 0 to 10 mM) for 24 h followed by washing. Cells were rested in RPMI medium for 5 days. On day 6, differentiated macrophages were restimulated with LPS 10 ng/mL for 24 h, and TNF‐α was measured in the supernatants, right: TNF‐α concentration measured by ELISA at day 7 of culture (mean + SEM, *n* = 6, *p < 0.05, Wilcoxon signed‐rank test).

To determine the involvement of the glutaminolysis pathway in trained immunity induced by oxLDL, we utilized a well‐established in vitro protocol for trained immunity in human monocytes (Figure 2A) [15]. Freshly isolated monocytes were exposed to oxLDL for 24 h. Following this, the monocytes were washed and allowed to rest and differentiate for a period of 5 days, thus becoming macrophages. On the sixth day, the oxLDL‐trained macrophages were restimulated with LPS for another 24 h. During the initial 24 h of oxLDL‐ or RPMI‐exposure, we inhibited the glutaminase enzyme using a pharmacological inhibitor, CB‐839 [29] (Figure 2A). Lactate dehydrogenase (LDH) and flow cytometry apoptosis assays demonstrated no evidence of cellular toxicity (Figure S2A–D). After a 24‐h treatment period, the inhibitor effectively blocked the conversion of glutamine to glutamate, thereby reducing the intracellular glutamate concentration within the monocytes (Figure 2B). Furthermore, CB‐839 co‐administration during the first 24 h of oxLDL exposure resulted in diminished TNF‐α production in oxLDL‐trained cells following LPS restimulation (Figure 2C). To further investigate the role of glutamine and its processing in oxLDL‐induced trained immunity, we induced trained immunity in monocytes in the presence of different concentrations of L‐glutamine during the initial 24‐h oxLDL exposure (Figure 2D). The absence of L‐glutamine did not prevent the establishment of the oxLDL‐induced trained immunity phenotype in vitro. Furthermore, the inhibition of one of the glutamine transporters, SLC1A5, with V9302 inhibitor [30] during the oxLDL treatment did not affect the cytokine production capacity and cell viability (Figure S3). Altogether, these data demonstrate that the conversion of glutamine to glutamate significantly contributes to the induction of oxLDL‐trained immunity, although glutamine is not indispensable.

### 3.3 Glutaminolysis Pathway Inhibition Prevents oxLDL‐Induced Increase in Energy Metabolism

**FIGURE 3:**
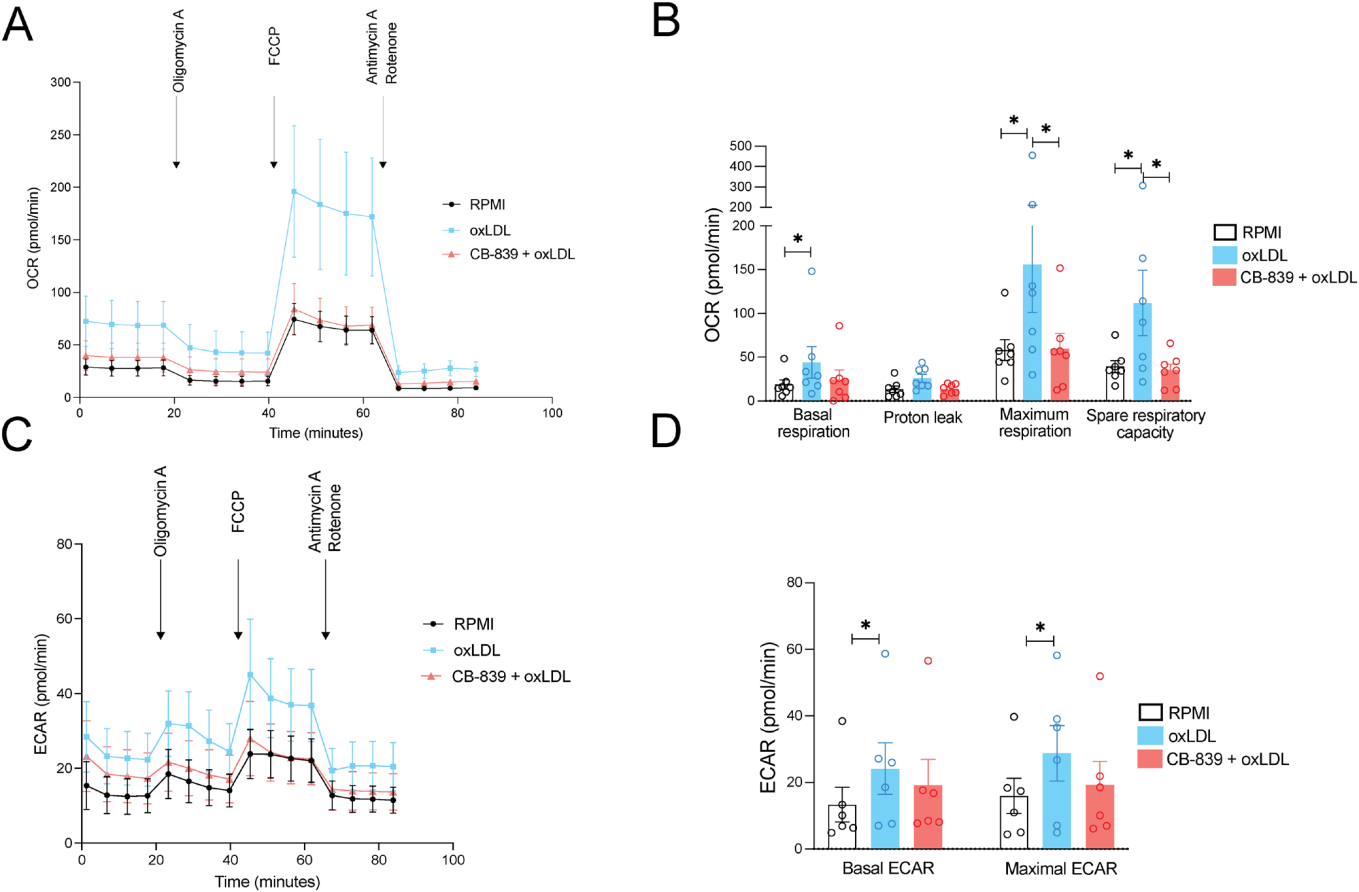
Glutaminolysis pathway inhibition prevents oxLDL‐induced increase in energy metabolism. (A) Oxygen consumption rate (OCR) of cells treated as in Figure 2A was determined by Seahorse XF technology at day 6 (before exposure to LPS). The data are shown as mean + SEM, *n* = 6. (B) Alterations in basal respiration, proton leak, maximal respiration, and spare respiratory capacity between different treatment conditions. One‐way ANOVA multiple comparisons test, *n* = 6, *p < 0.05, **p < 0.01. (C) Extracellular acidification rate (ECAR) of cells treated as in Figure 2A was determined by Seahorse XF technology at day 6 (prior to restimulation). The data are shown as mean + SEM, *n* = 6, *p < 0.05, Wilcoxon signed‐rank test. (D) Alterations in basal and maximal (Max) ECAR between treatment conditions as in a. One‐way ANOVA multiple comparisons test, *n* = 6, *p < 0.05, **p < 0.01.

Glutaminolysis functions as an anaplerotic pathway replenishing TCA cycle intermediates. OxLDL‐trained macrophages are characterized by increased glycolysis and oxidative phosphorylation [14, 24]. We subsequently determined the effect of glutaminolysis pathway inhibition on energy metabolism in oxLDL‐trained macrophages. To this end, we treated the monocytes with CB‐839 (Figure 2A), collected the oxLDL‐trained macrophages on day 6 of culture before LPS restimulation, and assessed metabolic assays to measure both mitochondrial and glycolytic activity (Figure 3). Consistent with previous findings [24], Seahorse XF analysis demonstrated that oxLDL‐trained cells exhibited augmented mitochondrial respiration (OXPHOS) measured by the OCR (Figure 3A,B). The increase in OCR in oxLDL‐trained cells was prevented by the inhibition of glutaminolysis. Furthermore, oxLDL‐treated cells showed increased basal and maximal extracellular acidification rate (ECAR), indicative of enhanced glycolysis, which was slightly reduced by CB‐839 (Figure 3C,D). Taken together, these findings suggest that glutaminolysis inhibition impairs oxLDL‐induced functional hyperresponsiveness by preventing the metabolic rewiring of monocytes.

### 3.4 oxLDL Exposure Enhances the Processing of Glutamine by the TCA Cycle

**FIGURE 4:**
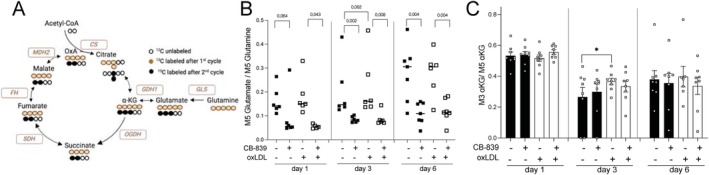
CB‐839 reduces glutaminase activity, while TCA cycle activity remains unaffected. (A) Schematic representation of atomic transitions in the TCA cycle metabolism from the [U^13^C] glutamine tracer. (B) Ratio of M5 glutamate to M5 glutamine determined during monocyte differentiation as indicated in Figure 2A. The cells were labeled with [U‐13C]‐glutamine and treated with RPMI or oxLDL in the presence or absence of the CB‐839 inhibitor. Numbers on the y‐axis signify the number of ^13^Carbons incorporated. (C) Ratio of M3 to M5 α‐ketoglutarate (α‐KG) measured by gas chromatography–mass spectrometry during monocytes differentiation as indicated in Figure 2A. Mean + SEM, *n* = 8, *p < 0.05, Wilcoxon signed‐rank test.

To further comprehend how glutaminolysis fuels the TCA cycle during trained immunity and the manner through which this is affected by glutaminolysis pathway inhibition, intracellular fluxes were measured using gas chromatography‐mass spectrometry (GC–MS) (Figure 4A). To this end, monocytes were labeled with a [U‐^13^C]‐glutamine tracer. Briefly, the monocytes were differentiated according to the protocol described in Figure 2A, and 24 h before harvesting, cells were placed in culture media containing [U‐^13^C]‐glutamine tracer.

The activity of the enzyme glutaminase (GLS) was determined by the ratio of labeled glutamine carbons incorporated into glutamate (M5 glutamate/M5 glutamine). This appeared to be slightly higher in the oxLDL‐exposed cells, with a *p*‐value of 0.06 at the 3‐day time point (Figure 4B), which is consistent with the observation that *GLS* expression is higher during the initial period after oxLDL exposure (Figure 1D). Furthermore, this measurement confirmed the effect of inhibition of glutaminase by CB‐839. Interestingly, there was a persistent inhibition of glutaminase on days 3 and 6 (Figure 4B). This finding suggested irreversible glutaminase inhibition by CB‐839. To exclude that the inhibitory effect of CB‐839 exposure in the first 24 h of the trained immunity protocol on cytokine production (Figure 2C) is due to persistent glutaminase inhibition throughout the macrophage differentiation period, we performed additional experiments in which we only added CB‐839 inhibitor on day 3 of the oxLDL‐trained immunity protocol, and we did not observe any decrease in TNF‐α production upon LPS restimulation (Figure S4).

**FIGURE 5:**
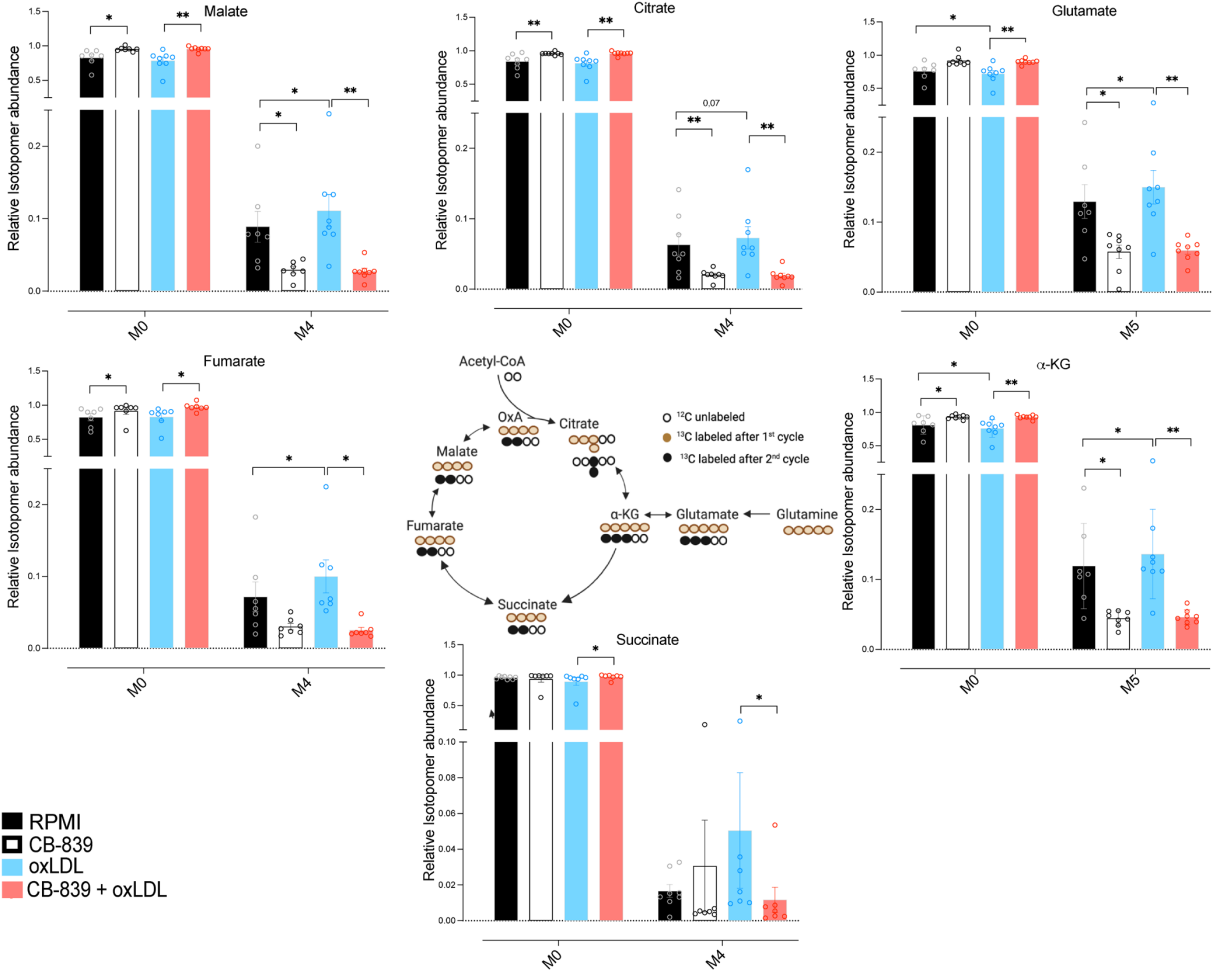
oxLDL treatment increases the processing of glutamine to TCA cycle metabolites, and this is prevented by glutaminolysis inhibition. Relative mass isotopomer distribution (MID) of TCA cycle metabolites in macrophages treated as in Figure 2A, labeled with [U‐^13^C]‐glutamine at day 2 and assessed at day 3 of culture. Numbers on the x‐axis signify the number of ^13^Carbons incorporated. Data are shown as mean + SEM, *n* = 7, *p < 0.05, **p < 0.01, Wilcoxon signed‐rank test.

Subsequently, we investigated the impact of glutaminase inhibition on TCA cycle activity. To do so, the utilization of glutamine throughout the TCA cycle was measured as the ratio of the α‐KG mass isotopomers produced during the first cycle (M3 α‐KG) and the initial fully enriched α‐KG (M5 α‐KG) (Figure 4A). The results of this study revealed that there was no effect of neither oxLDL nor CB‐839 on TCA cycle activity in trained monocytes during the first 24 h (Figure 4C). This suggests that the functional effects of oxLDL and CB‐839 on cytokine production are not due to changes in TCA cycle activity within the first 24 h. Interestingly, an augmentation in TCA cycle activity was observed in oxLDL‐treated macrophages on day 3 of culture (Figure 4C). We subsequently measured the intracellular concentrations of TCA cycle intermediates and observed that neither oxLDL exposure nor CB‐839 affected the concentrations of these intermediates at 24 h. Interestingly, we did observe a trend for intracellular accumulation of fumarate in oxLDL‐treated cells at day 3, which was prevented by co‐incubation with CB‐839 during the first 24 h (Figure S5). To further investigate the TCA cycle activity at this particular time point, we determined the utilization of glutamine into the metabolites of the TCA cycle, measuring their mass isotopomer distribution (MID) (Figure 5). As expected, in oxLDL‐treated macrophages, there is an enrichment of labeled carbons derived from glutamine in metabolites like glutamate, α‐ketoglutarate, fumarate, and malate over the RPMI control (Figure 5). Conversely, when glutaminase is inhibited in oxLDL‐trained macrophages, there is a reduced glutamine utilization into glutamate and all TCA cycle metabolites. Altogether, these results clearly show how brief exposure to oxLDL changes the glutamine replenishment of the TCA cycle: although during the 24 h of oxLDL exposure, there is no increase in this process, glutamine usage in the TCA cycle is clearly upregulated at the day 3 time point. This is prevented by co‐incubating the cells with CB‐839 in the first 24 h.

### 3.5 Genetic Variants in Glutaminolysis‐Related Genes Affect oxLDL‐Induced Trained Immunity

**FIGURE 6:**
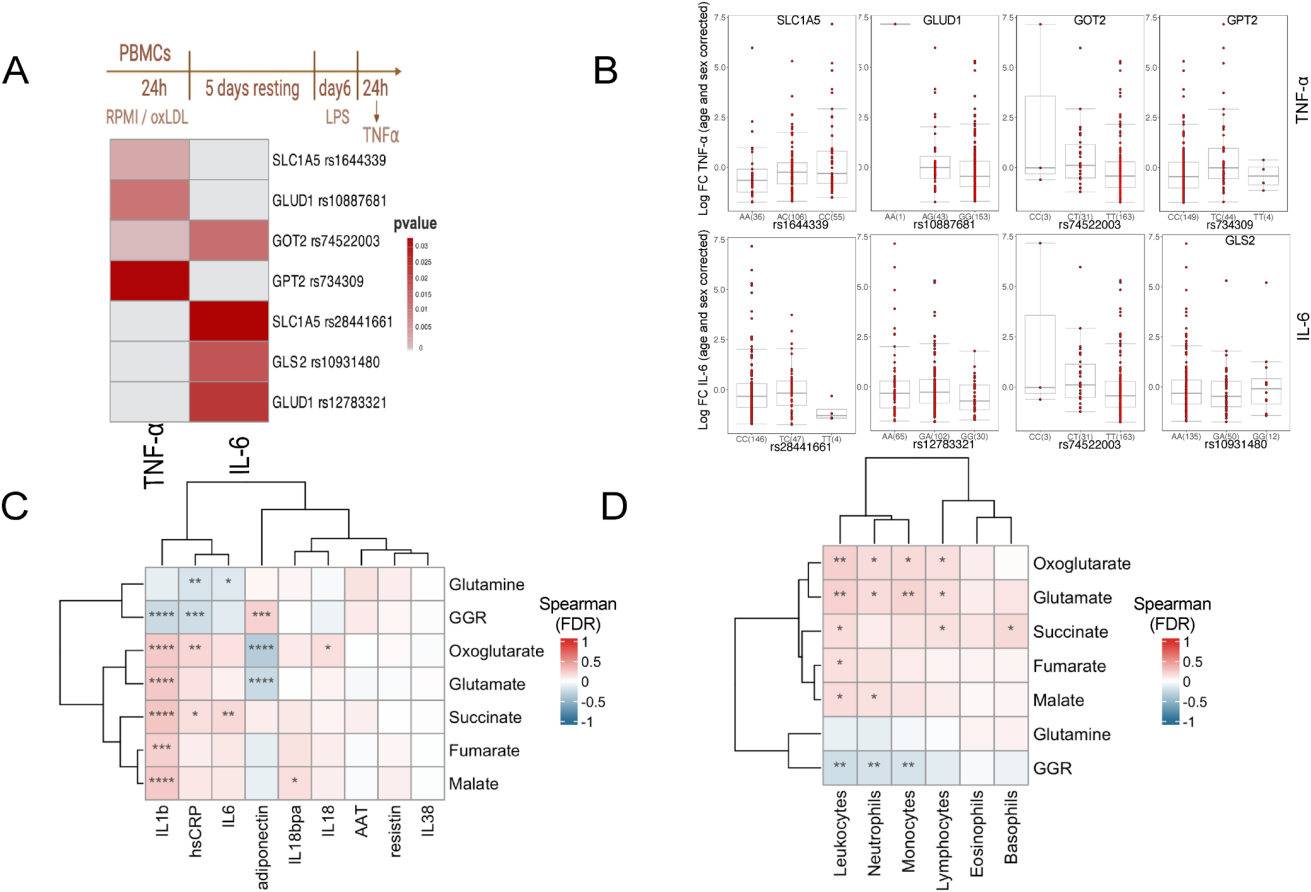
Glutaminolysis‐related genes and metabolites showed significant associations with markers of inflammation in human cohorts. (A) Heatmap of the *p* values for associations between SNPs mapped around genes (±150 kb) involved in glutaminolysis and the magnitude of TNF‐α and IL‐6 production capacity by PBMCs trained ex vivo with oxLDL (*n* = 243 healthy volunteers) [31]. (B) Age‐ and sex‐corrected TNF‐α and IL‐6 changes are shown as boxplots for selected SNPs in genes (±150 kb) involved in the glutaminolysis pathway, with the individuals being according to their genotype. Boxplots illustrate the median, upper, and lower quartiles. (C and D) Spearman correlations were calculated between plasma metabolites and inflammatory markers (C) and absolute count of circulating blood populations (D) measured in 302 obese patients from the 300 obese cohort (previously described [32, 33]). The heatmaps display the correlation coefficients, representing the strength and direction of the associations. Statistical significance was determined using the cor.test function in R, with adjustments for multiple tests performed using the false discovery rate (FDR). Asterisks denote significance levels: *p < 0.05, **p < 0.01, ***p < 0.001, and ****p < 0.0001.

In order to further validate the essential role of the glutaminolysis pathway in oxLDL‐trained immunity, genetic analyses were performed on a previously described cohort of 243 healthy volunteers [31]. In all these individuals, oxLDL‐induced trained immunity was assessed in ex vivo trained PBMCs (according to the same protocol as depicted in Figure 2A) and expressed as fold change of TNF‐α and IL‐6 production compared to cells that were exposed to only RPMI during the first 24 h (Figure 6A). We have now conducted a comprehensive evaluation of common SNPs (MAF > 0.05) within a 150‐kilobase window surrounding the glutaminolysis‐related genes: *SLC1A5, GLS2, GLUD1, GOT2*, and *GPT2*. We subsequently assessed the effects of these SNPs on cytokine production capacity by the oxLDL‐trained PBMCs (Figure 6A,B). The analysis identified various SNPs within 150 kilobases of the aforementioned genes that showed an association with a P nominal significance (*p* < 0.05) with alterations in TNF‐α and IL‐6 production. We found significant correlations between SNPs in close proximity to *SLC1A5* (rs1644339, *p* = 0.004), *GLUD1* (rs10887681, *p* = 0.013), *GOT2* (rs74522003, *p* = 0.001), and *GPT2* (rs734309, *p* = 0.034) genes with an enhanced TNF‐α production by PBMCs following in vitro oxLDL‐induced trained immunity. *SLC1A5* (rs28441661, *p* = 0.035), *GLS2* (rs109311480 *p* = 0.019), *GLUD1* (rs12783321, *p* = 0.025), and *GOT2* (rs74522003, *p* = 0.015) SNPs were found to be associated with enhanced IL‐6 production. These observations confirm our pharmacological data on GLS inhibition that the glutaminolysis pathway is indeed important in regulating oxLDL‐induced trained immunity formation.

### 3.6 Circulating Concentrations of Metabolites Linked to Glutaminolysis Correlate With Systemic Inflammatory Proteins in Obese Individuals

Because our results indicate that glutaminolysis is involved in oxLDL‐induced trained immunity, and previous studies showed that trained immunity affects inflammation and atherogenesis in experimental atherosclerosis [24, 34, 35], we further explored whether glutaminolysis affects systemic inflammation in humans at risk of developing atherosclerosis. Therefore, we performed an exploratory study using data from a previously described cohort comprising 302 individuals with overweight and obesity [32, 33]. We made use of metabolomics data, and selected metabolites related to glutaminolysis and the TCA cycle, and we used data on circulating markers of inflammation. The metabolites were measured in the plasma of these volunteers and we calculated associations of these metabolites with proinflammatory markers (Figure 6C) and immune cell frequencies in the circulation (Figure 6D). Our analysis focused on glutamine and metabolites relevant to glutamine usage in the TCA cycle such as glutamate, oxoglutarate (α‐ketoglutarate), succinate, fumarate, and malate. We also determined the glutamine‐to‐glutamate (GGR) ratio as it has been shown to be associated with cardiovascular health [36, 37, 38, 39, 40].

First, IL‐1β plasma concentrations were positively associated with all metabolite concentrations apart from glutamine and negatively correlated with GGR (Figure 6C). Second, IL‐6 concentrations are positively associated with succinate levels. IL‐18 concentrations were positively associated with oxoglutarate (α‐ketoglutarate) abundance, and IL‐18 binding protein alpha (IL‐18bpa) was positively associated with malate. Third, C‐reactive protein (CRP), a biomarker commonly associated with vascular inflammation and endothelial dysfunction [41], showed positive correlations with succinate and oxoglutarate and negative correlations with GGR. Conversely, plasma IL‐6 and CRP levels showed an inverse correlation with circulating glutamine. Fourth, positive correlations have been found between TCA cycle metabolite concentrations in the plasma and frequencies of circulating immune cells (Figure 6D). For example, we observed a positive association between leukocyte count and glutamate and all TCA cycle metabolites measured. Finally, GGR negatively correlated with leukocyte, neutrophil, and monocyte frequencies. Of particular note is the positive association of the monocyte absolute count with glutamate and oxoglutarate. Increased monocyte count, particularly intermediate and non‐classical monocytes, have been observed to be associated with the progression of atherosclerosis and cardiovascular disease (CVD) [42]. Altogether, these data indicate an association between glutamine processing by TCA cycle and the proinflammatory status of obese individuals.

## 4 Discussion

Innate immune cells can build a de facto immunological memory, and this functional adaptation of monocytes has recently emerged as one of the mechanisms contributing to the development of atherosclerosis [3]. To exploit this pathophysiological target for the design of new preventive strategies against CVD, it is critical to elucidate intracellular molecular mechanisms that drive the development of trained immunity. While the glutaminolysis pathway is needed for the establishment of β‐glucan‐induced trained immunity [4], its role in oxLDL‐trained immunity remains to be elucidated. In the present study, we demonstrated using a combination of pharmacological and genetic strategies that the glutaminolysis pathway is critical for the formation of oxLDL‐induced trained immunity. We showed that in oxLDL‐exposed cells, there is a rapid increase in RNA expression of *GLS* and *SLC1A5* in the first 24 h. After 3 days, glutaminase activity and glutamine replenishment of the TCA cycle increased with an accumulation of fumarate. Inhibition of glutaminase, the enzyme responsible for the conversion of glutamine to glutamate, impeded glutamine anaplerosis, prevented the increase in oxygen consumption rate, and compromised TNF‐α production by oxLDL‐trained cells. Altogether, these findings suggest that glutaminolysis is essential to build a trained immunity phenotype after short‐term oxLDL exposure.

Blocking glutaminase with pharmacological inhibitors prevented the hyperinflammatory‐trained immune phenotype. Interestingly, blocking the glutamine uptake by inhibiting the ASCT2 transporter did not affect the oxLDL‐induced trained immunity phenotype. Probably, this is due to the fact that ASCT2 is not the sole glutamine transporter but that several solute carriers regulate amino acid homeostasis [43]. We also showed that in vitro glutamine supplementation during the oxLDL exposure was not necessary to induce the trained immunity phenotype and that higher than physiological levels of glutamine did not further boost trained immunity. In this experiment, we did not measure the intracellular metabolite fluxes to investigate whether glutamine supplementation resulted in increased glutaminolysis. We conclude that in the presence of glutamine, glutaminolysis contributes to trained immunity development, although suppletion with hyperphysiological amounts of this amino acid did not further enhance trained immunity.

In our studies, we used CB‐839 [44] to inhibit glutaminase in monocytes. The inhibitor is classified as a non‐competitive inhibitor [29]. Surprisingly, using ^13^C labeled isotope, we showed that glutaminase activity still effectively decreased on days 3 and 6 after the exposure to CB‐839 during only the first 24 h (Figure 4B). With this observation, it was critical to exclude that this ongoing glutaminase inhibition throughout the macrophage differentiation in the trained immunity protocol prevented trained immunity, instead of the inhibition during only the 24 h exposure to oxLDL. Indeed, when we added CB‐839 only on day 3 of the trained immunity protocol, the augmented cytokine production capacity that characterizes trained immunity was unaffected (Figure S4). This clearly showed that glutaminase activity is essential in the first 24–48 h after oxLDL exposure when the trained immunity phenotype is induced.

Our tracing experiment revealed that the TCA cycle increased the usage of glutamine, but we did not see a significant accumulation of TCA cycle metabolites in oxLDL‐trained cells. We only observed a significantly higher fumarate concentration on day 3 within the cells in comparison to the previously reported findings in β‐glucan‐induced trained immunity [4], where the metabolome analysis showed accumulation of succinate, fumarate, and malate on day 6. Furthermore, glutaminolysis inhibition significantly reduced only fumarate levels. Although fumarate alone can induce trained immunity phenotype [4], the glutamine processing by the TCA cycle might also play a role in the induction of trained immunity. Despite the augmentation in glutamine processing to glutamate (Figure 5) in oxLDL‐induced trained macrophages on day 3, we do not observe an increase of glutamate intracellular concentration (Figure 2B) in oxLDL‐trained cells at that time point, nor after 24 h stimulation, pointing out the rapid processing of this metabolite through TCA cycle.

Posttranscriptional histone modifications maintain the trained immunity phenotype [45], and these epigenetic changes can directly result from changes in intracellular metabolites, which can act as substrates or activity modifiers [46]. Fumarate, for example, regulates epigenetic remodeling during trained immunity through the KDM5 family of histone demethylases, which are responsible for the demethylation of histone (H) 3 lysine (K) 4 [4]. H3K4 trimethylation (me3) is an activating histone mark. Fumarate inhibits KDM5, which leads to the enrichment of H3K4me3 on the promoters of proinflammatory genes, increasing their expression. OxLDL‐induced trained immunity is also associated with increases in H3K4me3 at the promoters of proinflammatory genes in trained cells [6]. Further studies should investigate the impact of glutaminolysis on epigenetic processes involved in trained immunity.

We validated the conclusion that glutaminase activity impacted trained immunity development using a genetic strategy. Importantly, there is a considerable interindividual variation in the capacity of monocytes to build trained immunity, which is determined by both genetic and epigenetic processes [47]. We previously showed that the trained immunity response of isolated monocytes to oxLDL was also affected by genetic variation in genes of key proteins involved in glycolysis and OXPHOS pathways in oxLDL‐induced trained immunity [24, 31]. We have now conducted such an evaluation on a cohort of 243 healthy subjects and identified a significant association between SNPs in close proximity to genes encoding for key proteins involved in glutamine handling and the cytokine production capacity of oxLDL‐trained cells. All of the significant gene variants found in glutaminolysis‐related genes are intronic variants that may affect gene transcription by altering transcription factor binding sites within regulatory regions—a key mechanism by which SNPs can influence complex traits and diseases [48]. Larger QTL studies on trained immunity are needed to investigate whether these genetic variants influence specific inflammatory pathways or cytokines. Our findings corroborate the pharmacological data, where glutaminolysis critically regulates oxLDL‐induced trained immunity.

Glutamine is a crucial energy substrate for rapidly dividing cells, and during inflammation, in order to meet their elevated energy requirements, macrophages exhibit augmented uptake of this amino acid [49]. Glutamine is processed through two main routes: oxidative and reductive carboxylation. Oxidative glutamine metabolism involves oxidation of α‐ketoglutarate to succinate and further to citrate by the TCA cycle, while reductive glutamine metabolism involves the conversion of α‐ketoglutarate to citrate. The enzyme glutaminase initiates both processes by deaminating glutamine into glutamate; thus, treatment with the CB‐839 inhibitor can affect both processes. Although the glutamine tracing experiment showed reduced incorporation of glutamine to citrate by reductive metabolism in the presence of the glutaminase inhibitor, we found no differences in reductive carboxylation between oxLDL‐treated and non‐treated cells (data not shown). This suggests a less important role of reductive glutamine metabolism in oxLDL‐induced trained immunity. These data are consistent with findings that the reductively metabolized glutamine is primarily used as a major carbon source for fatty acid synthesis in specific conditions, such as hypoxia and defective mitochondrial respiration [50, 51].

Considering the importance of glutaminolysis for trained immunity, which in turn contributes to systemic inflammation and atherosclerosis development, we performed an additional study to explore whether glutaminolysis‐related metabolites show an association with inflammation in patients at risk for CVD. In a previously well‐described cohort of 302 individuals with a BMI > 27 kg/m^2^ [32, 33], we found positive associations between plasma glutamate concentrations and circulating inflammatory markers and immune cell numbers, whereas glutamine and the glutamine‐to‐glutamate ratio (GGR) showed negative associations. Interestingly, several clinical trials identified high plasma glutamate concentrations and low GGR to be associated with unfavorable cardiometabolic status and cardiovascular mortality [36, 38, 39]. To our knowledge, this study is the first to report a negative correlation between the GGR and the proinflammatory status in individuals with obesity. The GGR was negatively correlated with circulating IL‐1β, CRP, leukocyte, neutrophil, and monocyte counts. Altogether, this suggests an interplay between glutamine metabolism and systemic inflammation in individuals at risk for atherosclerosis. A limitation of this exploratory study is that we do not know the source of the circulating metabolites in this study and the correlation with immune cell metabolism. It is known that the circulating glutamine and glutamate concentrations are determined, at least in part, by hepatic glutaminolysis. Recent studies have shown that the GLS isoform 2 (GLS2) is involved in this process [40]. GLS exists in two isoforms in mammals: GLS1, also called kidney‐type glutaminase (KGA) and GLS2 also called glutaminase B (GAB) and liver‐type glutaminase (LGA). Both isoforms are primarily expressed in mitochondria. GLS1 is widely distributed in most non‐liver tissues and various cells, including immune cells, while GLS2 is specifically expressed in the liver [52]. Previous studies describe opposing effects of GLS2 on atherogenesis [53]. In the study by Murcy et al., *GLS2*‐deficient *ApoE* ^−/−^ mice fed an atherogenic diet showed an increased atherosclerotic plaque area, accompanied by the recruitment of Ly6C^hi^ monocytes to the plaque, exacerbated inflammation, and lesion development [40]. In a similar knockdown experiment, Liu et al. observed a reduction in the plaque, improved lipidomic profiles, and decreased IL‐1β and IL‐6 levels in the aortic artery [53]. Therefore, the exact role of the GLS2 in atherogenesis remains to be elucidated. Future studies are required to explore underlying mechanisms and the relation between the circulating metabolites and glutaminolysis activity in immune cells.

In conclusion, we demonstrate that glutaminolysis critically regulates oxLDL‐induced trained immunity. This new finding complements recent murine studies showing an important role of hepatic glutaminolysis in atherosclerosis. Together, these findings suggest this pathway as an exciting and effective pharmacological target to prevent atherosclerotic cardiovascular diseases.

## Author Contributions

N.P.R., A.S., and K.P. conceived and designed the study and wrote the manuscript, with contributions from M.G.N., S.B., L.G., L.A.B.J., and K.H. A.S., J.Y, A.H., and T.M. performed the experiments. A.S., A.H., L.G., and T.M. acquired and analyzed the results. All authors critically read the manuscript.

## Conflicts of Interest

The authors declare no conflicts of interest.

## Supporting information


Figure S1.

## Data Availability

All data required to assess the findings presented in this study are included within the main text and the Supporting Information. The data can be provided by Prof. Niels Riksen upon reasonable request. Requests for the data should be submitted to: niels.riksen@radboudumc.nl.
