# Supplementary material for: Trained Immunity Induced by Oxidized Low‐Density Lipoprotein Is Dependent on Glutaminolysis
Source: FASEB J. 2025 Jun 27;39(13):e70774. doi: 10.1096/fj.202500802R (PMC12204306; doi:10.1096/fj.202500802R)
Supplement: Supplementary file 1 — Figure S1. [file FSB2-39-e70774-s001.docx]

**Supplementary Figures**

Supplementary Figure 1

**Supplementary Figure 1: *GLS2* is upregulated in MPP progenitors of patients with coronary artery disease (CAD).** Volcano plot showing differentially expressed genes (DE) in multipotent progenitor (MPP) population. Blue dots represent downregulated genes and red upregulated genes between patients with carotid artery disease (CAD) and individuals without. Highlighted is *GLS2* gene.

****Supplementary Figure 2

**Supplementary Figure 2: CB-839 does not induce cellular toxicity. a**) LDH release in conditioned media of monocytes incubated for 24h with RPMI or 50 uM CB-839 in the presence or absence of oxLDL treatment. **b**) Viability of the macrophages at day 6 of trained protocol, cells were stained for Live/Dead dye. Monocytes were incubated with medium alone (RPMI), 50 uM CB-839 inhibitor or trained with oxLDL in the presence of the inhibitor for 24 h followed by washing and recovery for 5 days. On day 6, macrophages were analysed by Flow Cytometry. The data are shown as mean + SEM, *n* = 7, **p* < 0.05, Wilcoxon signed-rank test. **c**) Apoptosis assessment upon treatment. The cells at day 6 were double-stained with annexin V-FITC and Live/dead dye. Graphs represent the percentage of apoptotic cells that are Annexin V^+^, Life/dead^-^. The data are shown as median + SEM, *n* = 6, **p* < 0.05, Wilcoxon signed-rank test.

**Supplementary Figure 3

**Supplementary Figure 3: Inhibition of glutamine transporter ASCT2 with V9302 inhibitor does not affect trained immunity induction by oxLDL.** **a**) TNF-α fold change to vehicle (DMSO). TNF-α was measured by ELISA in macrophage culture supernatants on day 6 after 24h restimulation with LPS treated as in **Figure 2A**. Monocytes were cultured with 500 nM and 10 µM of the V9302 inhibitor for the initial 24 hours of the trained immunity protocol. (median + SEM, n = 7, **p*<0.05, Wilcoxon signed-rank test). **b**) LDH release in conditioned media of monocytes incubated for 24h with RPMI or 500 nM and 10μM V9302 inhibitor in the presence or absence of oxLDL treatment.


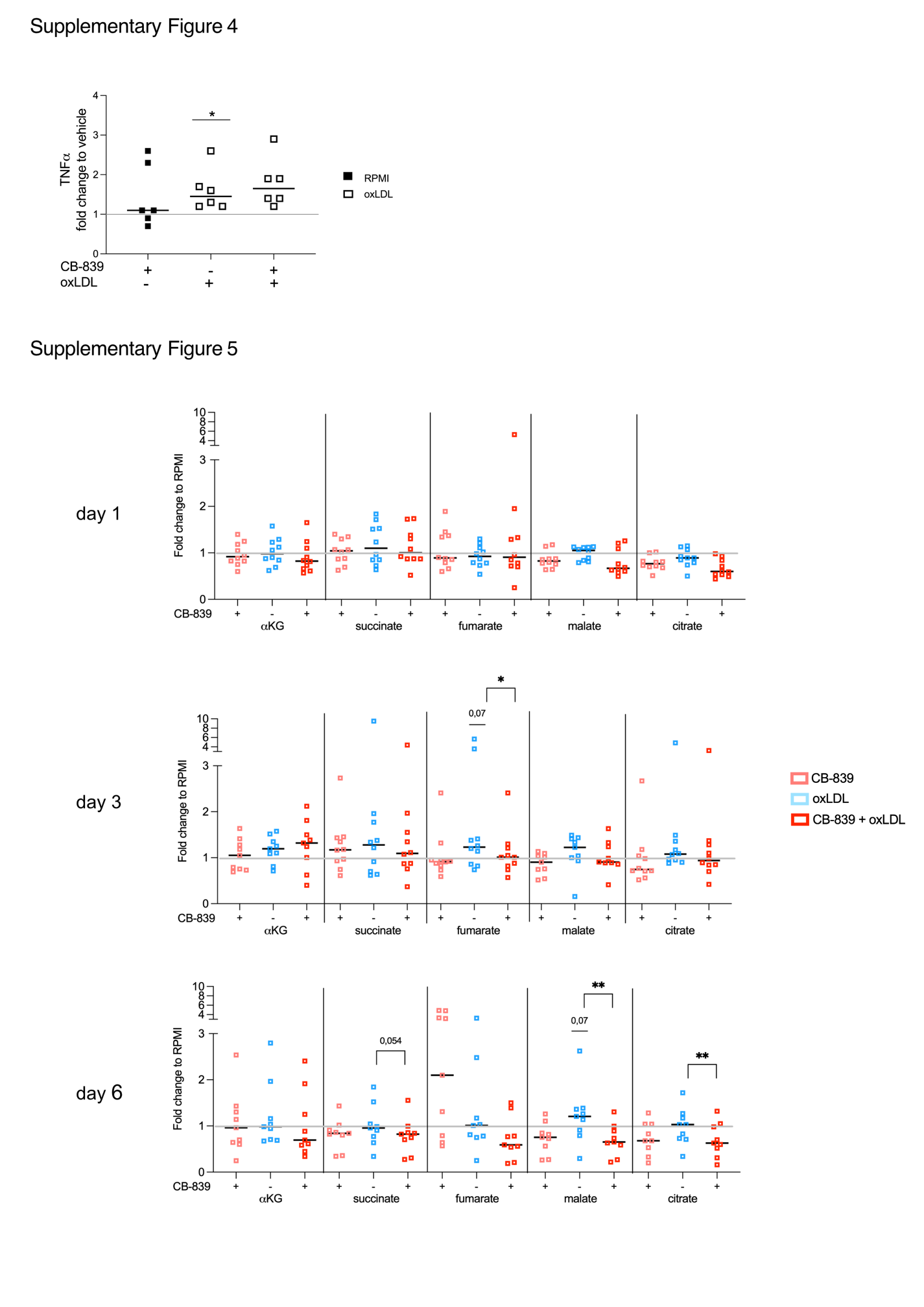
Supplementary Figure 4

**Supplementary Figure 4**: **Inhibition of GLS at day 3 does not affect the establishment of oxLDL-trained immunity.** TNF-α fold change to the vehicle control DMSO. TNF-α was measured by ELISA in macrophage culture supernatants on day 7 after 24h restimulation with LPS. In this experiment, GLS inhibitor was added to the culture on day 3 and washed out on day 6 before restimulation.

Supplementary Figure 5


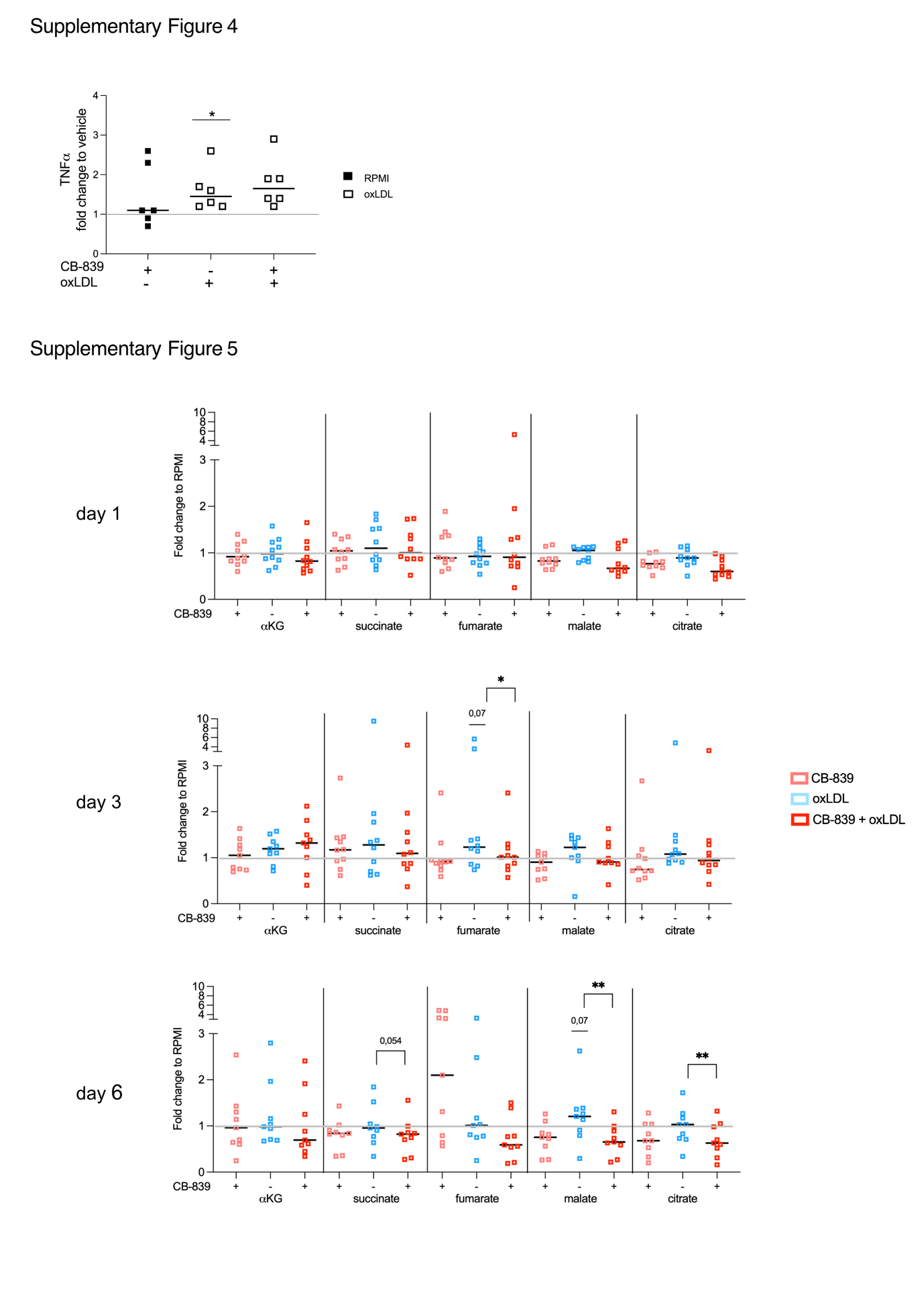


**
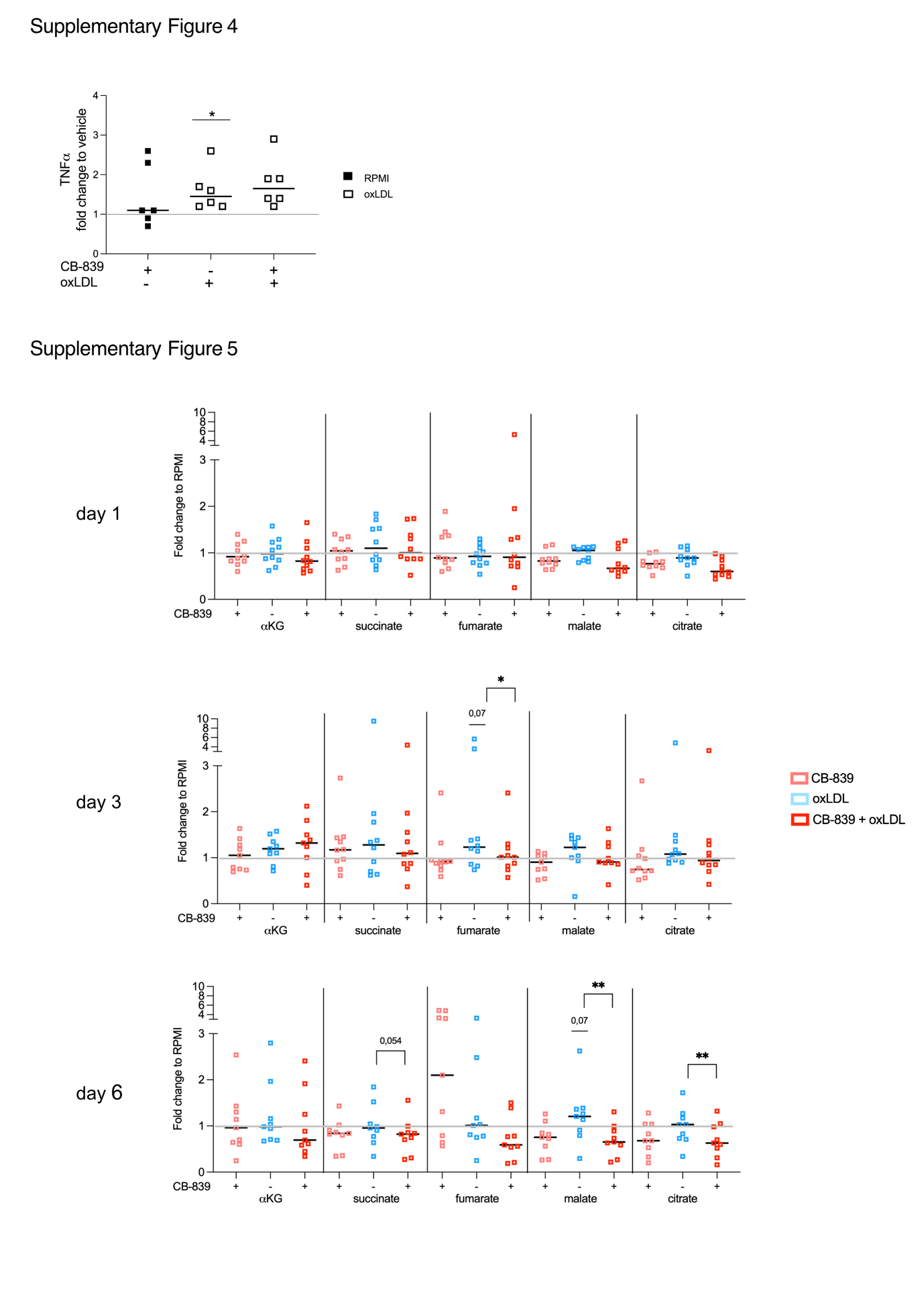
**

**
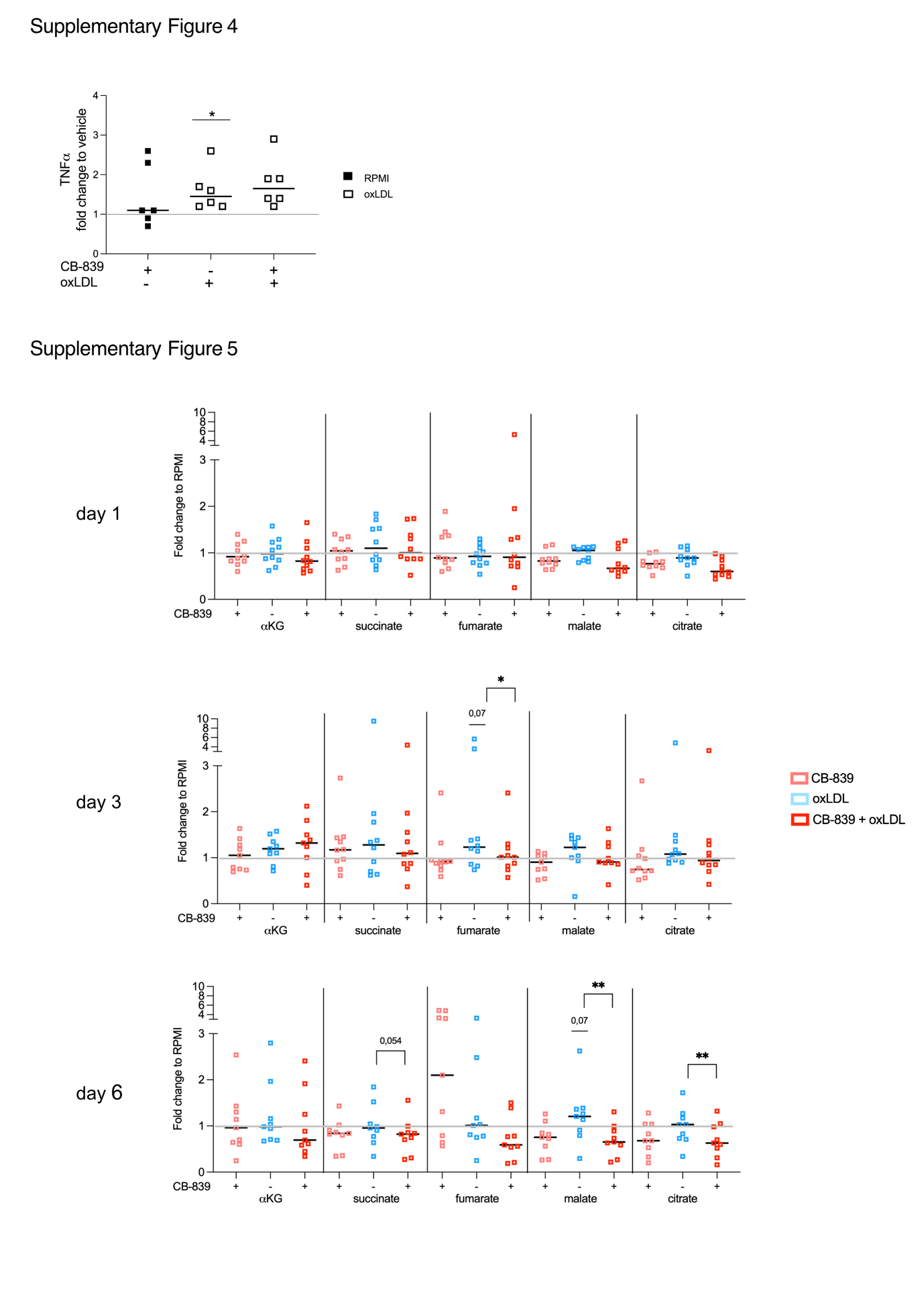
Supplementary Figure 5**: **Fumarate accumulation in oxLDL-treated macrophages.** Intracellular metabolites measured by gas chromatography-mass spectrometry, relative abundance to RPMI. Cells were collected and metabolites were measured at day 1, day 3 and day 6 of the protocol period **Figure 2A**. Data are shown as median + SEM, n = 7, **p*<0.05, Wilcoxon signed-rank test.
